# Genotyping of* Cryptosporidium* Species and Their Clinical Manifestations in Patients with Renal Transplantation and Human Immunodeficiency Virus Infection

**DOI:** 10.1155/2016/2623602

**Published:** 2016-02-14

**Authors:** Asmita Dey, Ujjala Ghoshal, Vikas Agarwal, Uday Chand Ghoshal

**Affiliations:** ^1^Department of Microbiology, Sanjay Gandhi Postgraduate Institute of Medical Sciences, Lucknow, Uttar Pradesh 226014, India; ^2^Department of Immunology, Sanjay Gandhi Postgraduate Institute of Medical Sciences, Lucknow, Uttar Pradesh 226014, India; ^3^Department of Gastroenterology, Sanjay Gandhi Postgraduate Institute of Medical Sciences, Lucknow, Uttar Pradesh 226014, India

## Abstract

In the present study we aimed to determine (i) frequency of* Cryptosporidium *species among patients with renal transplantation (RT) and human immunodeficiency virus (HIV) infection and (ii) relationship of the nature, severity, and duration of symptoms with different species and load of* Cryptosporidium*. Stool samples from 70 (42 RT and 28 HIV) and 140 immunocompromised patients with and without cryptosporidiosis by modified Kinyoun's staining were subjected to qPCR-melting curve analysis for identification of parasite species. qPCR detected one microscopically negative sample to be positive for cryptosporidiosis.* C. hominis*,* C. parvum*, and mixed infection were detected in 50/71 (70.4%), 19/71 (26.8%), and 2/71 (2.8%) patients, respectively. Patients with cryptosporidiosis had higher stool frequency (median, IQR: 4, 3–6/d versus 3, 2–4/d; *P* = 0.017) and watery stool (52/71 [73%] versus 64/139 [46%]; *P* = 0.003). Parasite load (median, IQR: Log_10_ 6.37 (5.65–7.12), Log_10_ 5.81 (4.26–6.65); *P* = 0.046) and nausea/vomiting (29/50 [58%] versus 5/19 [26%]; *P* = 0.032) were more frequent with* C. hominis *than with* C. parvum *infection. Thus,* Cryptosporidium *spp. (mainly* C. hominis*) is a common cause of diarrhoea in RT and HIV patients.

## 1. Introduction

The coccidian enteric parasite* Cryptosporidium* spp. is a common cause of gastroenteritis and diarrhoea in man [1, 2]. It causes severe, voluminous watery diarrhea in immunocompromised patients such as those infected with human immunodeficiency virus (HIV) [3, 4]. However, there is a paucity of studies on* Cryptosporidium* spp. among renal transplant (RT) recipients [5, 6]. Infected patients exhibit varying degrees of clinical manifestations. Some patients have severe symptoms over long duration while others recover in 1-2 weeks after a mild attack. The reasons for such diversity, which might be attributed to different genotypes or species of* Cryptosporidium* spp., or their varying load or host immunity, have not been adequately investigated so far.

Human cryptosporidiosis is mainly caused by* Cryptosporidium hominis* and* C. parvum*, which are responsible for most of the outbreaks of* Cryptosporidium* described so far. Other less common species are* C. meleagridis*,* C. cuniculus*,* C. viatorum*,* C. muris*,* C. canis*,* C. felis*,* C. suis*, and* C. andersoni* [7–13].* C. hominis* may cause more severe infection than* C. parvum* and other zoonotic species [7, 14–16]. In contrast, in a study from India, HIV positive patients, persons infected with* C. parvum* and other zoonotic species tended to have fever more frequently than those infected with* C. hominis* [11]. Thus, this variation in clinical manifestations might be due to the different* Cryptosporidium* species. Genotyping of* Cryptosporidium* has mainly been studied in patients with HIV infection. Currently there is no study on genotyping of* Cryptosporidium* spp. among renal transplant recipients. Also, relationship between nature, severity, and duration of the symptoms and different species of* Cryptosporidium*, among RT recipients, is not known.

An inverse relationship between occurrence of cryptosporidiosis and CD4 T cell count has been well documented in many studies in patients with AIDS. HIV/AIDS patients having lower CD4 count (<200 cells/cumm) are at higher risk of cryptosporidiosis [4, 17], with severe infections below 50 cells/cumm CD4 count [3, 18]. Such studies are scanty among RT recipients. In fact, the involvement of cell-mediated immunity in cryptosporidiosis has not been investigated in renal transplant recipients.

Microscopy and ELISA are the mainstay for diagnosis of* Cryptosporidium* spp. but are unable to differentiate among different species. Molecular technique like polymerase chain reaction-restriction fragment length polymorphism (PCR-RFLP) is the mainstay for genotyping. However, it is time consuming, requires post-PCR processing, and is prone to cross-contamination and false-positive results [19]. Real-time quantitative PCR (qPCR) is a highly sensitive and specific molecular tool which detects* Cryptosporidium* spp. and quantifies its load. Being a closed tube assay it prevents post-PCR contamination and obviates the need of time consuming procedures, like gel electrophoresis. The diagnostic efficacy of qPCR needs evaluation considering lack of adequate data.

Accordingly, we undertook a prospective study to determine (i) frequency of different species of* Cryptosporidium* infecting immunocompromised patients (HIV and RT patients) by real-time PCR (qPCR) and (ii) relationship of the nature, severity, and duration of the symptoms with different species of* Cryptosporidium* and their loads.

## 2. Materials and Methods

### 2.1. Patients

**Figure 1:**
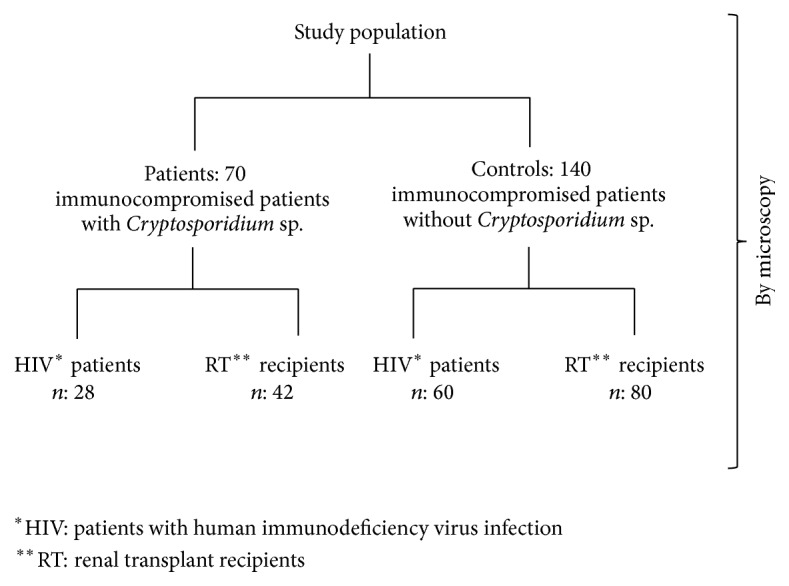
Flowchart showing study population and groups in the present study.

70 immunocompromised patients (42 RT recipients and 28 patients with HIV infection), having* Cryptosporidium* infection, with and without diarrhoea, attending a tertiary care centre, between 2008 and 2014, were included in this study. Patients with HIV infection were included as per guidelines of National AIDS Control Organization [20]. RT recipients undergoing at least one renal transplantation were recruited. Microscopy was used for detection and qPCR for genetic characterization of* Cryptosporidium* spp. Oocyst load was also determined by qPCR. Clinical and laboratory parameters of patients with cryptosporidiosis were compared with 140 immunocompromised controls (60 HIV and 80 RT recipients) without cryptosporidiosis (Figure 1). Data on demographic, clinical, and laboratory parameters were recorded in a standard questionnaire in each patient. The study protocol was approved by Institutional Ethics Committee (Ref. number PGI/PhD/IEC/57/21.10.2011) and it was in accordance with the Helsinki Declaration [21].

### 2.2. Sample Collection

Three consecutive stool samples from each patient and control were subjected to microscopy and culture. A part of it was stored at −40°C in normal saline for DNA extraction. Blood in EDTA vials was collected from the patients for determining the CD4 count of the patients.

### 2.3. Sample Processing

Stool samples were subjected to routine microscopy using saline and iodine direct wet mount technique for detection of cysts and trophozoites of parasites. After stool concentration by formal ether concentration technique, acid fast staining (modified Kinyoun's) was used for detection of oocysts of* Cryptosporidium* spp. Briefly, the smear was incubated in modified Kinyoun's stain for 20 minutes and malachite green was used as counter stain [22]. The stained smears were subjected to light microscopy. Stool samples were also subjected to culture on MacConkey, deoxycholate citrate agar (DCA), and dextrose sorbitol rhamnose agar (DSRA) to exclude other common bacterial agents causing diarrhoea such as* Salmonella* and* Shigella* spp. [23].

### 2.4. Genetic Characterization of* Cryptosporidium* spp. 

#### 2.4.1. DNA Extraction

Three consecutive stool samples were pooled into one aliquot from each patient and control and subjected to DNA extraction using QIAamp DNA-stool Mini-kit (QIAGEN Inc., Valencia, CA, USA) according to the manufacturer's protocol with some modifications; these included initial washing in 2% polyvinyl polypyrrolidone (PVPP) dissolved in 1x phosphate buffered saline (PBS) and incubation with stool lysis buffer (Buffer ASL) at 80°C for 10 minutes, with occasional stirring in between. DNA was purified and eluted as per manufacturer's instructions.

#### 2.4.2. Polymerase Chain Reaction (PCR)

Polymerase chain reaction (PCR) was performed using four primers for the amplification of dihydrofolate reductase (DHFR) gene, one sense and three antisense primers (CINF and CINR, 1R, 2R), aiming to detect* Cryptosporidium* spp.,* C. hominis*, and* C. parvum*, respectively. The primers used have been described previously [24]. Samples, positive for* Cryptosporidium* spp. but negative for the two genotypes, were sequenced. Primer-pairs CINF and CINR amplified a 575 bp region of the DHFR gene. Primer-pair CINF and 1R amplified a 357 bp region and primer-pair CINF and 2R, a 190 bp region of the DHFR gene. PCR was performed in a total volume of 20 *μ*L, consisting of 10 *μ*L of Green Taq Master Mix (2x), 10 pmoles of forward and reverse primers, and 1 *μ*L of template DNA. 100 ng/*μ*L of template DNA from each sample (extracted DNA) was used in PCR reactions. Three sets of reactions were performed with each sample; one with primer set CINF and CINR (for detection of* Cryptosporidium* spp.), second with primer set CINF and 1R (for detection of* C. hominis*); third with primer set CINF and 2R (for detection of* C. parvum*). PCR conditions were initial denaturation at 95°C for 5 min, followed by 40 cycles of denaturation at 94°C for 40 sec, annealing at 54°C for 30 sec, and elongation at 72°C for 45 sec. This was followed by a final elongation step of 72°C for 5 min. The PCR products were confirmed by sequencing.

Amplified products were subjected to electrophoresis in 2% agarose gel. Sequencing of the amplicons was performed using respective species-specific PCR primers. The sequences were compared with those available in the GenBank databases with the BLAST N program.

#### 2.4.3. Real-Time PCR

The amplification was carried out in real-time PCR machine Corbett Research 6000 Q-PCR instrument (Rotor-Gene 6000 software, Sydney, Australia) using the same sets of species-specific primers used in the traditional PCR [24]. The amplification round was carried out in a total volume of 20 *μ*L containing 10 *μ*L of KAPA-SYBR FAST Master Mix (Universal qPCR kit; Kapa Biosystems), containing KAPA-SYBR DNA Polymerase (an engineered version of Taq DNA Polymerase designed specifically for real-time PCR, SYBR Green I dye and optimized magnesium chloride concentration). Template DNA (having a concentration of 100 ng/mL per sample) was added. PCR conditions were as follows: initial denaturation at 95°C for 10 min, 40 cycles at 95°C for 45 sec, then annealing at 54°C for 60 sec, and extension at 72°C for 40 sec. Fluorescence data were collected in the extension step. For meting curve analysis (MCA), after completion of last PCR cycle, a quick denaturation was performed at 95°C for 1 sec, followed by a 30 sec annealing step at 45°C with a slow ramp (0.2°C/sec) up to 80°C. Reaction mixture without template DNA was used as negative control.

#### 2.4.4. Standard Graph

**Figure 2:**
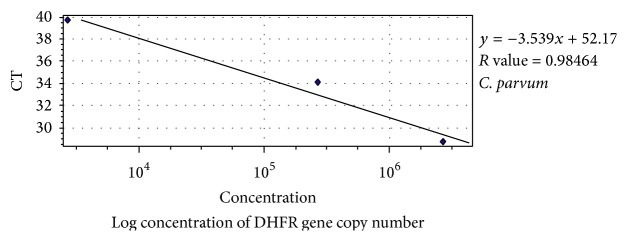
Standard graph (plotted with serially diluted DNA extracted from* Cryptosporidium parvum* oocyst).

Standard graph was plotted with DNA extracted from purified* Cryptosporidium parvum* oocyst, commercially purchased from The University of Arizona, USA. Oocysts (concentration of 5 × 10^7^ oocysts/mL in antibiotic solution) were subjected to DNA extraction by previously mentioned protocol using the QIAamp DNA Mini Kit (QIAGEN Inc., Valencia, CA, USA) [25, 26]. Standard graph was generated via serial dilution of oocyst stock (5 × 10^7^ oocysts/mL) to 5-fold (corresponding to approximately 10^7^ to 10^3^ oocysts/mL/dilution). The DHFR gene copies/*μ*L corresponded approximately to four times the oocysts/dilution due to the presence of single-copy of this gene/sporozoite or 4 copies/oocyst, as one oocyst consists of 4 sporozoites. DNA was extracted from each serial dilution and 100 ng/*μ*L of the extracted DNA was subjected to qPCR using primers specific for* C. parvum* to estimate the DHFR gene copy number of each serially diluted DNA and thus to construct the standard graph (Figure 2).

### 2.5. Data Analysis

Nonparametric and parametric continuous variables were analysed by Mann-Whitney *U* test and unpaired *t*-test, respectively. Categorical variables were analysed by Chi-square test. The values of equivalent oocyst concentrations (parasite load) were transformed into Log_10_ and presented as median and interquartile range (IQR). *P* values <0.05 were considered significant. All the statistical analyses were performed using Statistical Package for the Social Sciences (SPSS 15, Inc., Chicago, IL, USA).

## 3. Results

### 3.1. Demographic, Clinical, and Laboratory Parameters of Patients and Controls

70 immunocompromised patients were positive for* Cryptosporidium* spp. by both microscopy and qPCR and one by qPCR alone. Though the oocyst load in the stool of this microscopically negative RT recipient, initially included in the immunocompromised control group, was comparatively lower, being log_10_ 6.65 equivalent oocyst concentration, he was considered as infected. Patients with other parasitic infestation and enteropathogenic bacteria were excluded from analysis. The demographic and clinical parameters of the 71 patients with cryptosporidiosis and 139 immunocompromised controls are shown in Table 1. Cryptosporidiosis was associated with diarrhoea, increased stool frequency, and watery stool (Table 1).

**Figure 3:**
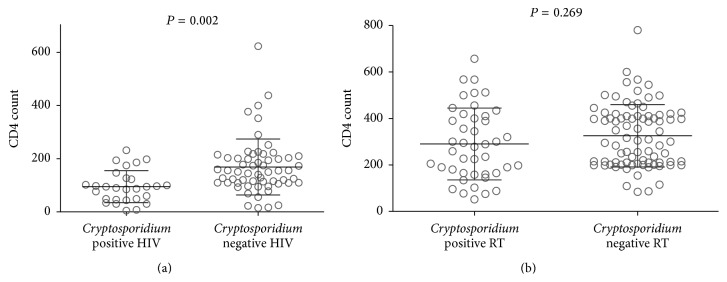
Comparison of CD4 counts of HIV patients with and without cryptosporidiosis; (b): comparison of CD4 counts of RT patients with and without cryptosporidiosis. The bar represents the average. Unpaired Student's *t*-test was used for comparison between groups. *P* value <0.05 was considered as significant. Values given in text (Section 3.1).

Immunocompromised (both HIV and RT) patients with cryptosporidiosis had lower CD4 count than noninfected ones (mean ± SD: 214 ± 162 cells/cumm versus 260 ± 144 cells/cumm, *P* = 0.041). CD4 count of patients with HIV infection was lower than patients with RT (145 ± 10.6 cells/cumm versus 313 ± 12.7 cells/cumm, *P* = 0.004). CD4 count of HIV-infected patients with cryptosporidiosis was lower than those without (95 ± 11.3 cells/cumm versus 169 ± 13.7 cells/cumm, *P* = 0.002) (Figure 3). In contrast, CD4 counts of RT patients, with and without cryptosporidiosis, were comparable (290 ± 23.6 cells/cumm versus 326 ± 15 cells/cumm, *P* = 0.269) (Figure 3). Moreover, CD4 count of HIV-infected patients with cryptosporidiosis was lower than that of* Cryptosporidium* infected RT patients (223 ± 23 cells/cumm versus 260 ± 12.3 cells/cumm, *P* = 0.09); neutrophil count in RT patients with cryptosporidiosis tended to be lower than that in* Cryptosporidium* infected HIV patients (67 ± 10.5 cells/cumm versus 72 ± 14 cells/cumm, *P* = 0.08).

The RT recipients with cryptosporidiosis more often received triple immunosuppressive drugs than those without, who more often received either double or single immunosuppressants (41/43, 95.4% versus 2/43, 4.6% versus 0/43; *P* = 0.013). Mycophenolate mofetil (MMF), Wysolone, and Tacrolimus were used in patients both with and without cryptosporidiosis (17/43, 39.5% versus 30/80, 37.5%; *P* = 0.85). History of antiretroviral treatment was reported more often by patients without than those with cryptosporidiosis (21/59 versus 3/29, *P* = 0.012).

### 3.2. Genetic Characterization of* Cryptosporidium* spp. 

**Figure 4:**
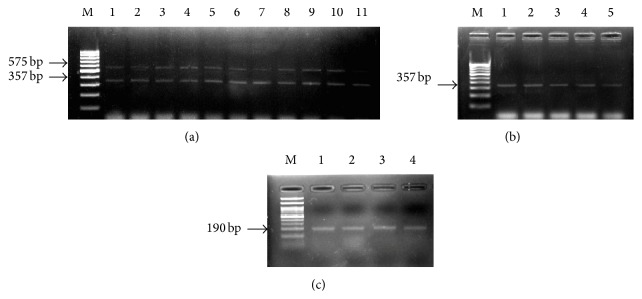
(a) Lane 1: multiplex polymerase chain reaction (PCR) of* C. hominis* positive control (showing bands for both* Cryptosporidium* spp. [575 bp] and* C. hominis* [357 bp]); Lanes 2–11: multiplex PCR of samples positive for* C. hominis*; (b) Lanes 1–5: PCR of samples positive for* C. hominis* (357 bp); (c) Lanes 1–4: PCR of samples positive for* C. parvum*. In all figures, M: 100 bp molecular marker.

**Figure 5:**
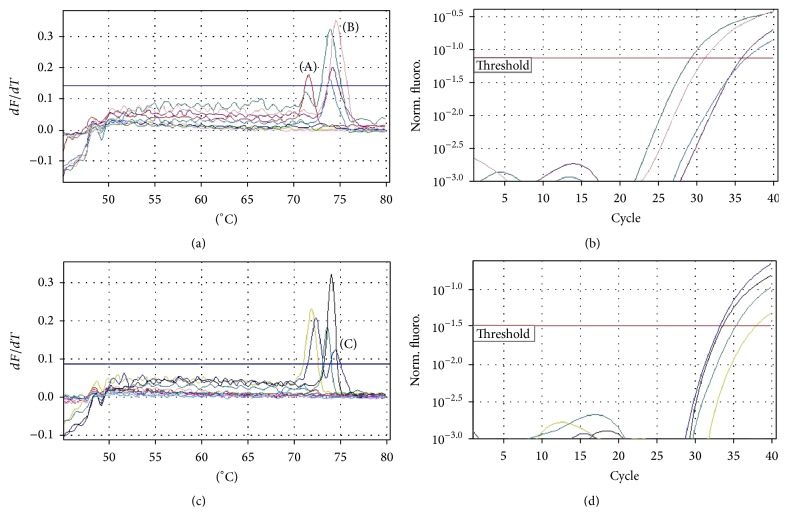
(a) and (c) Representative melting curves peaks; (b) and (d) respective amplification curves of samples; (A) melting curve peaks for samples positive for* C. parvum* (mean peak value: 72.6 ± 1.76°C); (B) melting curve peaks for samples positive for* C. hominis* (mean peak value: 74.8 ± 1.64°C); (C) melting curve peaks for samples positive for mixed infections with both* C. hominis* and* C. parvum*.

*Cryptosporidium hominis*,* Cryptosporidium parvum*, and mixed infection were detected in 50/71 (70.4%), 19/71 (26.8%), and 2/71 (2.8%) immunocompromised patients, respectively.* C. hominis*,* C. parvum*, and mixed infection were detected among 20/28 (71.5%), 7/28 (25%), and 1/28 (3.5%) HIV positive patients and 30/43 (69.8%), 12/43 (28%), and 1/43 (2.2%) RT recipients, respectively.* C. hominis* showed an average peak at 74.8 ± 1.64°C while* C. parvum* was at 72.6 ± 1.76°C, by MCA (Figures 4 and 5). Only one microscopically negative sample was positive for* C. hominis*, which was also confirmed by sequencing. The GenBank accession numbers of submitted sequences are BankIt1855902 Seq1, KT735247, BankIt1856029 Seq2, KT735248, BankIt1856031 Seq3, KT735249, BankIt1856033 Seq4, KT735250, BankIt1856035 Seq5, KT735251, BankIt1860090 Seq1, KT831947, BankIt1860090 Seq2, KT831948, BankIt1860090 Seq3, KT831949, BankIt1860090 Seq4, KT831950, and BankIt1860090 Seq5, KT831951.

### 3.3. Relationship between the Nature, Severity, and Duration of the Symptoms and Different Species

The demographic, laboratory, and clinical parameters of the patients infected with* Cryptosporidium hominis* and* C. parvum* are shown in Table 2. Patients with* C. hominis* infection more often had nausea and/or vomiting and increased oocyst load (denoted by equivalent concentration of oocysts in the stool samples) compared to patients infected with* C. parvum* (*P* < 0.05; Table 2). However, fever, abdominal pain, frequency, and duration of diarrhoea were comparable between patients with* C. hominis* and* C. parvum* infection (Table 2).

### 3.4. Relationship between the Nature, Severity, and Duration of the Symptoms and Parasite Load

The median load of oocysts of* Cryptosporidium* spp. in patients with HIV infection and those with RT was comparable (log_10_ 5.65 IQR (4.73–6.89) versus log_10_ 6.37 IQR (5.81–6.79), *P* = ns). Patients having diarrhoea had higher copy number (or equivalent oocyst concentration) of* Cryptosporidium* than those without (Log_10_ 6.37 IQR (5.8–7.05) versus Log_10_ 4.96 IQR (3.92–6.06), *P* = 0.03)).

## 4. Discussion

In the current study, we found that (a)* C. hominis* and* C. parvum* were the only two* Cryptosporidium* spp. detected in immunocompromised patients, (b) cryptosporidiosis was more commonly associated with diarrhoea, increased stool frequency, and watery stool, (c)* C. hominis* infection was more frequently associated with nausea and/or vomiting and increased oocyst load than* C. parvum*, and (d) patients with diarrhoea had higher copy number (or equivalent oocyst concentration) of* Cryptosporidium* spp. than those without.

In the present study,* C. hominis* and* C. parvum* were the only two* Cryptosporidium* spp. detected in immunocompromised patients. To date, genotyping of* Cryptosporidium* spp. has been done mostly on HIV-infected patients. There are scanty data on genotyping of* Cryptosporidium* spp. among RT recipients. In studies on HIV-infected patients from South Africa [27], North America [28, 29], South America [7, 30], and Europe [31],* C. hominis* was the most prevalent species. However, in a few other studies,* C. parvum* was found to be more prevalent than* C. hominis* [32–35]. Studies from India also revealed* C. hominis* to be the most prevalent species infecting HIV-infected patients [36, 37]. A recent study from northern India reported two of three* Cryptosporidium* isolates from RT recipients to be* C. hominis* and one* C. parvum* by PCR-RFLP for small subunit (SSU) rRNA gene [38]. This indicates that human is the major source of transmission of the infection. To the best of our knowledge, the present study is perhaps the only one from India, which used real-time PCR for detection and genetic characterization of* Cryptosporidium* spp.

In this study qPCR detected* Cryptosporidium* in one sample that was missed by microscopy. The load of oocysts in this sample was comparatively lower than the rest. Hence, stool microscopy done by an expert is as good as qPCR for detection of* Cryptosporidium*. However, qPCR is useful when oocyst load is less. In addition, species identification, which may have clinical implications, can be done by qPCR and is not possible by microscopy [36, 39]. qPCR has the advantage of real-time detection of PCR amplicons omitting the requirement of restriction digestion or sequence analysis for genotyping.

The current study shows that CD4 count was less in HIV patients with* Cryptosporidium* infection than without. It has been well documented in previous studies that patients having lower CD4 count are at higher risk of cryptosporidiosis. Chronic cryptosporidiosis generally occurs in patients with CD4 counts below 200 cells/cumm [18], and patients with CD4 count below 50 cells/cumm suffer from severe infections [3]. In fact, several studies have documented a correlation between CD4 count <200 cells/cumm and symptomatic cryptosporidiosis, in HIV patients [11, 17]. However, such studies are scanty in RT recipients. In our study, CD4 count among RT patients was not as low as that in HIV positive patients with cryptosporidiosis, suggesting that depletion of immune cells and factors other than CD4 count is responsible for susceptibility to cryptosporidiosis in RT patients. Subgroup analysis revealed neutrophil count tended to be lower in* Cryptosporidium* infected RT patients than in HIV patients. Low neutrophil count of RT patients might be attributed to some immune-suppressive drugs as well. Thus, not only immune restoration in the form of HAART therapy, but also management of immune-suppressive therapy may help in clearance of the parasite, in immunocompromised hosts.

In this study, the RT recipients with cryptosporidiosis were given mostly triple immunosuppressive drugs than double and single immunosuppressants. The combination of mycophenolate mofetil (MMF), Wysolone, and Tacrolimus drugs was mostly used in both patient and control groups. In a study from Pakistan, the immunosuppressive drugs administered to RT recipients having cryptosporidiosis comprised of cyclosporine/FK506, azathioprine/mycophenolate mofetil (MMF), and steroids (Prednisolone/Wysolone) [40]. In another study cyclosporin A has been used in infected RT recipients [41]. Till date no association of* Cryptosporidium* infection has been shown with any immunosuppressive drug. In fact, there are scanty data on cryptosporidiosis in RT recipients. Large-scale follow-up studies, explaining the type and dose of immunosuppressive agents and their management in* Cryptosporidium* infected RT recipients, could clarify this issue further.

In the present study,* C. hominis* was associated with frequent nausea and/or vomiting and increased oocyst load compared to* C. parvum*. Thus, infection caused by* C. hominis* was more severe than* C. parvum* infection. This is in accordance with the previous studies from Peru, which showed that* C. hominis* was associated with diarrhea, nausea, vomiting, general malaise, and increased oocyst shedding intensity and duration. In contrast, zoonotic isolates like* C. parvum* were associated with diarrhea only [7]. In a study from United Kingdom, duration of* C. parvum* infection was significantly lower than that of* C. hominis* (*F* = 8.312, *P* = 0.005) [14]. Also,* C. hominis* was reported to cause more severe infection than* C. parvum* [14]. It has been mentioned in this study that* C. hominis* being a “specialist,” particularly infecting the human host, show more virulent characteristics than a “generalist” like* C. parvum*. From Brazil, children with* C. hominis* infection have been reported to shed significantly more oocysts in stool [42]. Similarly in a study on children from India,* C. hominis* infection was associated with a greater severity of diarrhea [16]. In contrast, in a study from India on HIV positive patients, persons infected with* C. parvum* and other zoonotic species tended to have fever more frequently than those infected with* C. hominis* [11]. It is suggested that immunodeficiency is responsible for alteration of host susceptibility to the* Cryptosporidium* species that are normally noninfectious to humans [11].

The present study has a few limitations. The study does not involve follow-up of the patients. Follow-up studies on the same will determine the actual epidemiology of the infection and heterogeneity of clinical manifestations. Our findings, however, add some new information to the existing knowledge.

In conclusion,* Cryptosporidium* spp. is a common cause of diarrhoea in renal transplant recipients, as in HIV positive patients. Hence, clinicians should not overlook the possibility of occurrence of cryptosporidiosis in these patients.* C. hominis* is the most common* Cryptosporidium* spp. detected in both HIV and RT patients, suggesting that anthroponotic mode of transmission of the parasite is dominant in immunocompromised patients, particularly in northern India. Real-time PCR is an important and time-effective tool for diagnosis of* Cryptosporidium* spp. as well as its genetic characterization.

## Figures and Tables

**Table 1:** Demographic and clinical parameters of immunocompromised patients with and without infection with *Cryptosporidium* spp.

	Patients with *Cryptosporidium* infection (*n* = 71)	Patients without *Cryptosporidium* infection (*n* = 139)	*P* value
Age (mean, SD^*∗*^)	39 (13)	39 (11)	NS
Gender (male)	58/71 (81.7%)	115/139 (82.7%)	0.94
Duration of diarrhoea in days (median, IQR^*∗∗*^)	16 (3, 41)	15 (7, 60)	0.66
Diarrhoea	59/71 (83%)	84/139 (60.4%)	0.0014
Watery stool	52/71 (73%)	64/139 (46%)	0.0003
Daily stool frequency (median, IQR^*∗∗*^)	4 (3, 6)	3 (2, 4)	0.017
Fever	34/71 (48%)	65/139 (46.7%)	0.96
Cough	18/71 (26%)	41/139 (29.5%)	0.70
Nausea, vomiting	34/71 (49%)	59/139 (42.4%)	0.38
Abdominal pain	29/71 (41%)	56/139 (40.3%)	0.88
Drinking water (unfiltered)	9/41 (22%)	28/113 (25%)	0.717

(SD: Standard deviation, IQR: interquartile range and NS: not significant; Data of drinking water was available for only 41 patients with *Cryptosporidium* infection and 113 patients without).

**Table 2:** Demographic and clinical parameters between patients infected with *C*. *hominis* and *C*. *parvum*.

	Patients with *C*. *hominis* infection (50)	Patients with *C*. *parvum* infection (19)	*P* value
Age (mean ± SD) in years	40 ± 13	33.6 ± 13	0.43
Gender (males)	41/50 (82%)	16/19 (84.2%)	0.8
Melting curve (mean ± SD) °C	74.8 ± 1.64°C	72.6 ± 1.76°C	NS
Ct values (mean ± SD)	30.45 ± 3.43	30.5 ± 3.3	NS
Conc. (median, IQR)	Log_10_ 6.37 (5.65–7.12)	Log_10_ 5.81 (4.26–6.65)	0.046
Diarrhoea	41/50 (82%)	16/19 (84%)	1.00
Duration of diarrhoea (median, IQR)	19, 1–45	16, 3–60	0.89
Number of episodes (mean, SD)	5 ± 3.7	5 ± 2.8	0.45
Fever	23/50 (46%)	10/19 (52.6%)	0.77
Nausea/vomiting	29/50 (58%)	5/19 (26%)	0.032
Abdominal pain	22/50 (44%)	7/19 (37%)	0.95

(SD: Standard deviation, IQR: interquartile range, NS: not significant and Ct: cycle threshold, Conc.: Equivalent oocyst concentration (parasite load)).
